# Nuclear Receptor RORα/γ: Exciting Modulators in Metabolic Syndrome and Related Disorders

**DOI:** 10.3389/fnut.2022.925267

**Published:** 2022-06-21

**Authors:** Haotian Gu, Ping Hu, Yahui Zhao, Yaya Liu, Yi-Ting Wang, Abdelkareem A. Ahmed, Hao-Yu Liu, Demin Cai

**Affiliations:** ^1^College of Animal Science and Technology, Yangzhou University, Yangzhou, China; ^2^Department of Veterinary Biomedical Sciences, Botswana University of Agriculture and Agriculture and Natural Resources, Gaborone, Botswana; ^3^Biomedical Research Institute, Darfur University College, Nyala, Sudan

**Keywords:** RORα/γ, metabolic syndromes, sterols, insulin resistance, lipids

## Abstract

Under the influences of modern lifestyle, metabolic syndromes (MetS), including insulin resistance, obesity, and fatty liver, featuring a worldwide chronic disease, greatly raise the risk of type 2 diabetes, heart disease, and stroke. However, its pathogenesis is still unclear, and there are limited drugs with strong clinical efficacy and specificity. Given the close connection between impaired lipid metabolism and MetS onset, modulating the lipid metabolic genes may provide potential prospects in the development of MetS therapeutics. Nuclear receptors are such druggable transcription factors that translate physiological signals into gene regulation *via* DNA binding upon ligand activation. Recent studies reveal vital functions of the NRs retinoic acid's receptor-related orphan receptors (RORs), including RORα and RORγ, in the gene regulation in lipid metabolism and MetS. This review focuses on the latest developments in their actions on MetS and related metabolic disorders, which would benefit future clinically therapeutic applications.

## Introduction

Metabolic syndrome (MetS) is a cluster of conditions manifested as insulin resistance, obesity, fatty liver, and hyperlipidemia (1). The number of patients with MetS in the USA is more than one-third of the total population, while over 200 million Chinese adults suffer from it (2). As a complex condition, MetS conditions often occurs together and elevates the risk of type 2 diabetes, prevalence of stroke and myocardial infarction, and mortality rate of cardiovascular diseases, as well as compromise life quality and cause great economic burden (3). Given the primary pathological components in MetS, including impaired glucose and lipid metabolism, the current treatments of healthy lifestyles changes, including improved dietary plans, regular physical activity, reducing stress, and stopping smoking are helping, but not enough (4–6). For the purposes of drug discovery, nuclear receptors (NRs) like peroxisome proliferator-activated receptor (PPARs), liver X receptor (LXRs), REV-ERBs, and retinoic acid receptor-related orphan receptors (RORs) have favorable attributes, with most containing protected internal cavities predisposed for occupation with hydrophobic molecules of volumes typical of drugs. Moreover, these transcription factors control central pathways impacting a wide range of pathophysiological events ranging from cancers to metabolic diseases (7, 8).

The subfamily RORs (α, β, γ) has the typical NR domain structure involving a ligand-binding domain (LBD), along with a highly-conserved DNA-binding domain (DBD), an N-terminal domain, and a hinge domain spacing the DBD and LBD (9, 10). Accumulating evidence reveals that RORs as transcription factors exert the potential therapeutic capacities in numerous MetS-related metabolic diseases (11). In particular, the RORα and RORγ are closely associated with fatty acids and sterol metabolism, and their trans-activities could be driven by a series of sterols and synthetic ligands that function either as agonists or antagonists (12). In contrast, the expression of ROR-β is restricted to the brain and retina, and is commonly associated with retinal neurogenesis, etc. (13). These targetable characteristics provide the possibility of RORs-mediated therapeutic targets of metabolic illnesses. This mini-review aims at summarizing the connections between these orphan NRs and the onset of MetS and related disorders for better understanding of the underlying mechanisms in the process of pathogenesis. To explore the feasibility of using orphan NR ligands in the treatment of metabolic diseases according to published documents will benefit the theoretical strategies for further small-molecule drugs' development and clinical intervention.

## RORα Actions in Liver Pathophysiology and Adipose Tissue Homeostasis

Encoded by *NR1F1*, RORα is widely expressed in tissues like liver, lung, skin, kidney, adipose tissue, *etc*. (14, 15). RORα's full body knockout mice exhibit improved metabolic outputs on both chow and high fat diets, without overt behavioral dysfunctions (16). The natural RORα spontaneous mutant mice, *i.e*., the staggerer (sg) mice would be the initial evidence that RORα modulates metabolic processes by showing a lower expression pattern of genes involved in lipid metabolism, including apolipoprotein A-1 (apoA1) and apolipoprotein C-III (apoCIII) (17, 18). Thus, RORα^sg/sg^ mice display a lean body type compared to that of the wild-type mice. The sg mice perform the protective functions against diet-induced MetS and associated complications, such as fatty liver, adipose tissue inflammation, and glucose metabolic abnormalities (19). It is suggested that RORα plays a positive role in metabolic diseases by reducing oxidative stress and inflammation in livers when activated (20). Intriguingly, liver-specific RORα deletion (RORα^LKO^) in mice shows no significant effects on glucose and lipid metabolism in response to either chow or western-style diet (21). In contrast, RORα^LKO^ mice show the typical MetS including hepatic steatosis, obesity, and IR when supplemented with high fat diet (22). This is attributed to the RORα-mediated PPARγ transcriptional repression by HDAC3 recruitment. In agreement, Kim et al. also demonstrated that RORα^LKO^ mice are more susceptible to high-fat diet-induced non-alcoholic steatohepatitis (NASH) where RORα exerts a beneficial role in mitochondrial quality control by enhancing oxygen consumption rate and the expression of key genes *Bnip3* and *PGC-1*α (23). Same in this RORα deletion model, RORα inhibition induces suppression of hepatic polyploidization and hepatocyte DNA endoreplication to ameliorate the high fat diet (HFD)-triggered NASH, providing another insight of the protective mechanisms of RORα against MetS by maintaining genome integrity (24). In addition, RORα-targeting lysosomal acidification recovery is a new mechanism behind the HFD-induced NASH when RORα is knocked out (25). This is supported by several pieces of evidence, e.g., the lower lysosomal acidity, increased lysosomal translocation of the mechanistic target of rapamycin, accumulated immature cathepsin D, LC3-II, p62/sequestosome 1 (SQSTM1), and upregulated neighbor of BRCA1 gene 1 (NBR1) observed in the RORα^LKO^ mice. Importantly, the reduced key genes enrolled in lysosomal function in the livers of mice with HFD-caused NASH, and patients are activated by RORα. Regarding genes expression, a couple of genes involved in glucolipid metabolism, including phosphoenolpyruvate carboxykinase (Pepck), glucose-6 phosphatase (G6pc), glycerol-3-phosphate acyltransferase (G3pat), and perilipin 2 (Plin2) are downregulated in the RORα^−/−^ mice fed a HFD diet (26–28). In contrast, the sulfotransferase Sult1e1 and Sult2a1, as the sulfotransferase and bile acids sulfonation, are drastically upregulated in the RORα-deficient mice (29, 30). The genome-wide ChIP-seq analysis in the liver reveals that RORα directly binds to the ROR response elements at these glucolipid genes including *Pepck1, G6pc, Apoa1, Fasn*, and *Elovl3* to transcriptionally control their expression (31, 32).

It is worth mentioning that adipose tissues-associated inflammation is tightly linked to the development of MetS and related metabolic disorders (33, 34). RORα modulates insulin sensitivity and contribute to diminished inflammation in the RORα^−/−^ mice (35). Lipid droplets are seen to be reduced in the adipose tissue of RORα^−/−^ mice fed with an HFD, while decreased inflammatory macrophages infiltration and downregulated inflammatory genes are observed (26). Because of the potential role of interleukin-1 receptor antagonist (IL-1rn) in IR, the reduced Il-1rn expression in adipose tissue of RORα^−/−^ mice possibly leads to less susceptibility to MetS (36). Moreover, the neutral cholesterol ester hydrolase 1 (NCEH1), as a direct target of RORα, is required for RORα-mediated lipid droplets deposition in macrophage, and functions *via* a phorbol myristate acetate-dependent mechanism (37). To further define the potential role of RORα-expressing macrophages in the generation of an aberrant metabolic state, RORα^fl/fl^LysM^Cre/+^ mice, which do not express RORα in myeloid cells, were maintained on an HFD, and metabolic parameters were assessed (38). These mice had significantly impaired weight gain and improved metabolic parameters in comparison to RORα^fl/fl^ control mice. Further analysis of the immune cell populations within white adipose tissue deposits demonstrates a decrease in inflammatory adipose tissue macrophages (ATM). It is noted that endoplasmic reticulum (ER) stress and unfolding protein response (UPR) signaling play a critical role in inflammatory response in obese adipose tissue (39). Liu et al. revealed that RORα is also involved in this process, that RORα is potently induced by inflammatory stimuli in macrophages and is positively regulated by the inflammatory response by activating ER stress and UPR response (40). In contrast to adipose tissue, RORα exhibits the protection against NASH *via* enhancing M2 polarization in liver macrophages, while myeloid-specific knockout RORα are susceptible to HFD (41). Interestingly, it is also documented that RORα deficiency, which is specifically in macrophages, does not affect the development of IR, obesity, and NASH (42). This suggests a different Cre or Lyz2 copy numbers, but not a specific effect of RORα deletion in macrophages causes the impairments on NASH.

## RORγ is Closely Linked to Sterols' Metabolism

The RORγ is another key player in the modulation of MetS-related metabolic diseases. In this regard, insulin sensitivity and glucose tolerance were observed along with decreased glucose content in the liver of RORγ^−/−^ mice (43–45). This may be attributed to the suppression of hepatic gluconeogenesis and blunted transformation to glucose from pyruvate to glucose (43, 45). Indeed, ectopic RORγ activation in the RORγ^−/−^ mice or RORγ-deficient primary hepatocytes facilitates the glucose production recovery by improving the rate of gluconeogenesis (43). Consistent with the output, key genes involved in glucolipid metabolism, including *G6pc, Pklr, Gck, Glut2, Gckr, Ppar*δ, *Insig2a, Elovl3*, and *Sult1e1*, are remarkably upregulated compared to those of RORγ^−/−^ mice (30, 43, 44, 46). Given that Insig2a and Elovl3 are critical for insulin signaling and directly driven by RORγ, the restoration of these genes by exogenous RORγ over-expression support the RORγ-mediated MetS by ameliorating IR (47, 48). A genome-wide cistrome analysis of mouse liver reveals that RORγ directly binds to promoters and/or the enhancers of genes enrolled in gluconeogenesis and glycolysis, *e.g., G6pc, Pepck, Gckr, Pklr, Glut2, Gck, Gys2, Pcx*, and *Klf15* (44, 49). Thus, the downregulated transcripts of these genes in the RORγ^−/−^ mice are accompanied by impaired glucose generation. Importantly, the Insig2 repression is an indicator of activated lipogenesis in an SREBP1-dependent negative feedback loop; loss of RORγ in mice will disrupt the dynamic balance of lipid metabolism (48, 49). This would result in reduced contents of triglycerides in liver and blood with enhanced hepatic lipogenic gene expressions in RORγ-deficient mice when fed with HFD.

It is noted that cholesterol and bile acids were also reduced in liver and blood when Insig2 expression was lower in the RORγ null mice (46, 49). Indeed, RORγ modulates MetS more likely *via* regulating sterols' metabolism. The early finding of RORγ-mediated cholesterol metabolism should be attributed to the RORγ isoform 2, RORγt, which is mainly expressed in immune cells and is required for Th17 differentiation (12, 50). During this process, the key genes involved in cholesterol dynamic metabolism, including *Hmgxs1, Hmgcr, Lss, Dhcr24, Fdft1, Cyp7a, Cyp27a1, Abca1*, and *Abcg1*, were all affected (51). Given that squalene synthesis of mevalonate pathway is critical for cholesterol production, squalene supplementation is shown to improve RORγ-mediated transactivation in *in vitro* validation experiment. It is, therefore, expected that this transactivation was inhibited when the key gene *Fdft1* enrolled in squalene biosynthesis was deficient (52). In mammals, RORγ-controlling sterols' metabolism is intrinsically associated with the typical transcription factors like sterol regulatory element binding protein 2 (SREBP2) and liver X receptor (53–56). The increased intermediates like zymosterol and desmosterol involved in SREBP2-enhanced cholesterol biosynthesis *via* upregulating the genes, which encode the rate-limiting enzymes involving HMGCR and HMGCS, have been demonstrated to activate the sensitivity to RORγ (53, 54, 57). It is documented that RORγ directly binds to cholesterol biosynthetic genes, and positively regulates these genes over SREBP2 in a breast cancer cell line (58, 59). Intriguingly, RORγ is found to share the binding peaks with SREBP2 and determines the SREBP2 recruitments on the target genes. In the *in viv*o model using piglets, ChIP-seq analysis provides the proof that hepatic RORγ exhibited strong enrichments on the cholesterol biosynthesis genes at the enhancer region (60). In the liver organoids, RORγ agonists, such as desmosterol and a commercial drug SR0987, displayed drastical restoration of reduced cholesterol content, along with biosynthetic genes caused by a time-restricted feeding (61). Moreover, ectopic RORγ overexpression performed similar effects with those of the agonists possibly *via* epigenetically recruited histone active marks H3K27ac. A recent study also revealed RORγ as an essential mediator in facilitating the interaction of PPARα and SREBP2 and in controlling the cholesterol biosynthesis genes transcription (62). This new complex of “PPARα-RORγ-SREBP2” explored a new mechanism on cholesterol reprogramming in a context and cell-type-specific manner, which would be beneficial for the invention of therapeutic strategy for MetS and related metabolic diseases. Indeed, PPARα plays a novel role in MetS because of the changed functions induced by RORγ in different scenarios, suggesting the complicated crosstalk among these NRs (62, 63). Given the fact that LXRs modulate the cholesterol intake and efflux by the key enzymes such as LDLR and ABCA1/G1, it is suggested that a diminished RORγ availability is associated with decreased cholesterol production in response to LXR activation (53, 55, 64). Recent studies provide a pivotal clue that the derivative of lithocholic acids (LCA), 3-oxoLCA, is a novel inhibitor of RORγ (65, 66). Because the cholic acids are synthesized from cholesterol and are mainly modulated by nuclear receptor FXR, it is likely that RORγ is enrolled in the regulation of cholesterol-bile acid circulation and, maybe, a new mechanism to maintain cholesterol homeostasis against MetS. It is worth addressing that circadian rhythm exerts critical roles in MetS and RORα/γ are primary modulators of clock genes expression. This RORα/γ-driven circadian clock gene regulation is therefore another type of events controlling the biological balance against MetS and related disorders (7, 67).

## Conclusions and Perspectives

**Figure 1:**
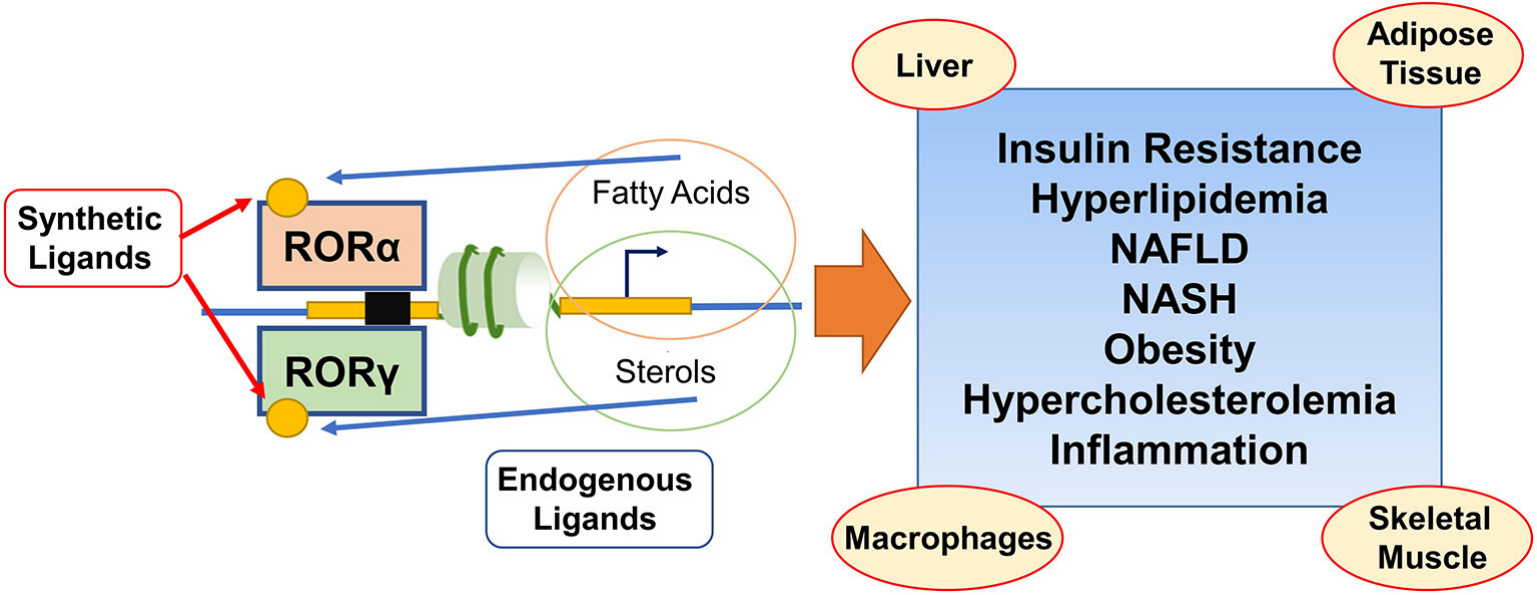
Schematic illustration depicting that coordinated actions of RORα/γ on lipid and sterol metabolic programming during the development of the metabolic syndrome and related disorders. Both RORα and RORγ regulate the target genes involved in lipid/sterol metabolism through ROR response elements and are exciting modulators in metabolic syndrome and related disorders in multiple tissues. RORs (ant)agonists from endogenous or synthetic generation, act as the potential prosects in the treatment of insulin resistance, hyperlipidemia, NAFLD, NASH, obesity, hypercholesterolemia, and inflammation.

Tremendous advances have been made in studying the underlying mechanisms in the pathogenesis of MetS and related metabolic diseases in recent decades. However, the pivotal targetable controllers are still being explored to find a therapeutic strategy. An increasing number of reports have provided evidence that RORα/γ exert numerous actions on lipid metabolism of MetS, as summarized in Figure 1. Given these NR transcriptional factors governing the development and progress of MetS, further studies are needed to understand the precise molecular mechanisms behind their crosstalk, and how alteration of NR function affects these interactions and MetS pathologies. Importantly, unlike the established master regulator of several metabolic pathways, RORα/γ exhibit diversity and plasticity of RORs functions and actions in a context and subtype-specific manner. These explorations of untapped RORs' features are worthy of further investigations on their biological importance. Elucidating the remaining intricacies will subsequently afford viable approaches for treating MetS. Even though current efforts to target the MetS-related pathogenesis have failed to develop efficient therapeutic agents, researchers in this field have gained substantial insights from the cases of those that have been available for use by targeting modulators and drivers like RORs in animal models of disease for the past. Furthermore, identification of endogenous high-affinity or synthetic ligands of RORs α/γ will not only increase our understanding of their actions on MetS and related disorders, but also probe the therapeutic potential of these receptors as bona fide drug targets for the treatments.

## Author Contributions

DC and H-YL: conceptualization. HG, PH, YZ, YL, and Y-TW: writing original draft. PH, H-YL, AA, and DC: review, editing, and resources. All authors contributed to the article and approved the submitted version.

## Funding

This work was supported by the Postgraduate Research & Practice Innovation Program of Jiangsu Province (SJCX21_1619), National Natural Science Foundation of China (32002243), Natural Science Foundation of Jiangsu Province (BK20200932), Natural Science Foundation of the Higher Education Institutions of Jiangsu Province (20KJB230001), the Jiangsu Agricultural Science And Technology Innovation Fund [CX(21)2014 and CX(21)3125], and the Priority Academic Program Development of Jiangsu Higher Education Institutions (PAPD).

## Conflict of Interest

The authors declare that the research was conducted in the absence of any commercial or financial relationships that could be construed as a potential conflict of interest.

## Publisher's Note

All claims expressed in this article are solely those of the authors and do not necessarily represent those of their affiliated organizations, or those of the publisher, the editors and the reviewers. Any product that may be evaluated in this article, or claim that may be made by its manufacturer, is not guaranteed or endorsed by the publisher.
